# Culture-Negative Endocarditis Complicated by Cerebral Abscesses Due to Streptococcus gordonii: A Diagnostic Odyssey

**DOI:** 10.7759/cureus.70775

**Published:** 2024-10-03

**Authors:** Dinesh Nirmal, Bernard Brown, Noah Silverstein, Andrea Trimmingham, Samy I. McFarlane

**Affiliations:** 1 Internal Medicine, State University of New York (SUNY) Downstate Health Sciences University, Brooklyn, USA

**Keywords:** cerebral abscess, culture-negative endocarditis, karius, septic emboli, streptococcus gordonii

## Abstract

Endocarditis is a serious diagnostic entity that carries a high rate of morbidity and mortality, with complications including heart failure, septic embolization, brain abscesses, and stroke. Blood culture-negative endocarditis (BCNE) represents a particularly challenging clinical scenario where the causative organism is undetectable, either due to being difficult to culture or due to the empiric administration of antimicrobial agents. This entity generally results in delayed diagnosis and treatment of endocarditis, with a potential increase in the rate of complications. In this report, we present a case of multiple brain abscesses resulting from BCNE, where the causative organism - and hence effective treatment - was only identified with the implementation of modern molecular diagnostic techniques like Karius, isothermal amplification methods, etc. We also highlight the specific entities of BCNE, its pathogenesis, and differential diagnosis, as well as the effective diagnostic and therapeutic options available to date.

## Introduction

Blood culture-negative endocarditis (BCNE) poses significant diagnostic challenges, representing approximately 2-7% of all endocarditis cases [1,2]. The inability to detect organisms in routine blood cultures is often due to prior antibiotic administration, which is responsible for 35% to 74% of culture-negative endocarditis (CNE) cases. Among the fastidious microorganisms, the *Haemophilus* species, *Aggregatibacter actinomycetemcomitans*, *Cardiobacterium hominis*, *Eikenella corrodens*, and *Kingella kingae* (HACEK) group, as well as nutritionally variant streptococci, are notable causes. Fungi account for approximately 1-2% of endocarditis cases, with *Candida albicans* being the most common fungal cause. Intracellular pathogens like *Bartonella* species and *Tropheryma whipplei* also contribute to CNE cases.

Complications of bacterial endocarditis are numerous and severe, with septic emboli occurring in 22% to 50% of cases. One particularly serious complication is the development of cerebral abscesses, which occur in approximately 2% to 10% of infective endocarditis cases. These abscesses can lead to significant neurological deficits, seizures, and increased intracranial pressure [3].

This case study explores a complex instance of CNE complicated by cerebral abscesses in a 67-year-old male, where *Streptococcus gordonii* was identified as the causative agent through advanced molecular diagnostics after traditional methods failed [4,5].

## Case presentation

This is a case of a 67-year-old male with a history of diabetes mellitus, hypertension, and atrial fibrillation; he has had multiple embolic strokes over the past seven years and a complex, evolving history of multiple complicated infections over the past two years. He presented with a two-week history of abdominal pain associated with altered mental status.

**Figure 1:**
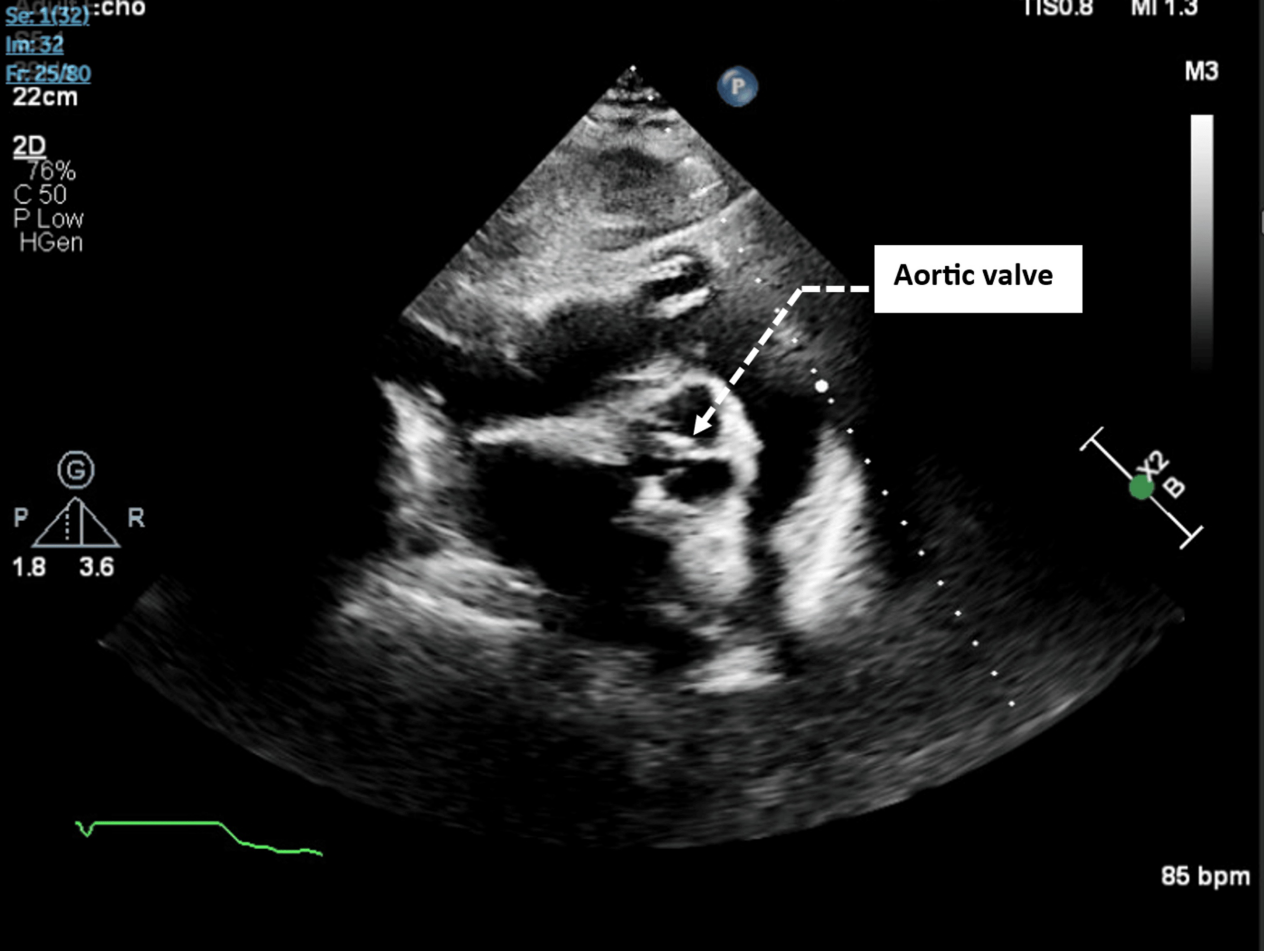
A transthoracic echocardiogram obtained at presentation to the hospital shows a seemingly normal aortic valve, with no visible vegetations.

A year prior to presentation, the patient had been admitted for evaluation of abdominal pain and fever in the setting of an indwelling urinary catheter and was subsequently diagnosed with a urinary tract infection (UTI) complicated by a *Candida tropicalis* fungal ball in the urinary bladder, associated with *Candida auris* fungemia. An implantable cardioverter defibrillator (ICD) placed two years prior was removed due to fungemia. The fungal ball in the bladder was endoscopically removed, followed by 14 days of continuous bladder irrigation with amphotericin deoxycholate. The patient was also started on systemic caspofungin for the fungemia and fluconazole for the treatment of the fungal ball, given the poor bladder penetration of echinocandins. Caspofungin was continued for six weeks after the last negative blood culture, which occurred on day 8 of hospitalization. Fluconazole was continued for a total course of four weeks. The patient was intermittently started on systemic amphotericin B, which had to be discontinued after two doses due to nephrotoxicity. The echocardiograms obtained during the hospital stay did not show vegetation or significant valvular abnormalities (Figure 1). The patient was subsequently discharged home after the completion of the antibiotic course.

**Figure 2:**
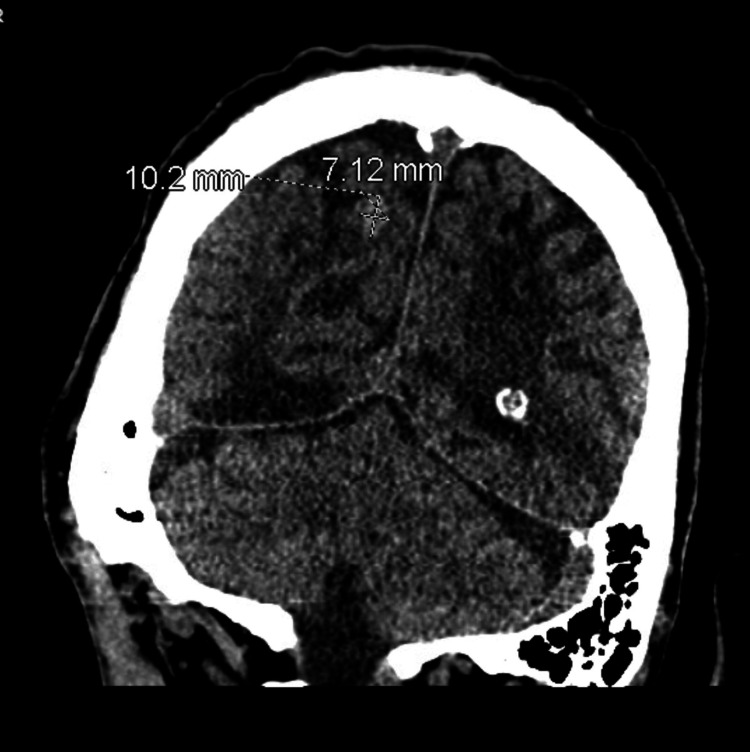
Computed tomography of the brain on day 1 of hospitalization: right parietal intraparenchymal hemorrhage measuring approximately 7 x 10 mm.

Subsequently, the patient presented with a two-day history of abdominal pain and right-sided weakness, with the baseline motor function being unclear. Physical examination elicited generalized abdominal tenderness, with no guarding or rigidity, and multiple neurologic deficits with an unclear baseline exam. Labs were significant for gross pyuria, with 1,047 white cells per high-power field, and the computed tomography (CT) head revealed a 10 mm right parietal intraparenchymal hyperdensity, likely an intraparenchymal hemorrhage (Figure 2).

Clinical course

**Figure 3:**
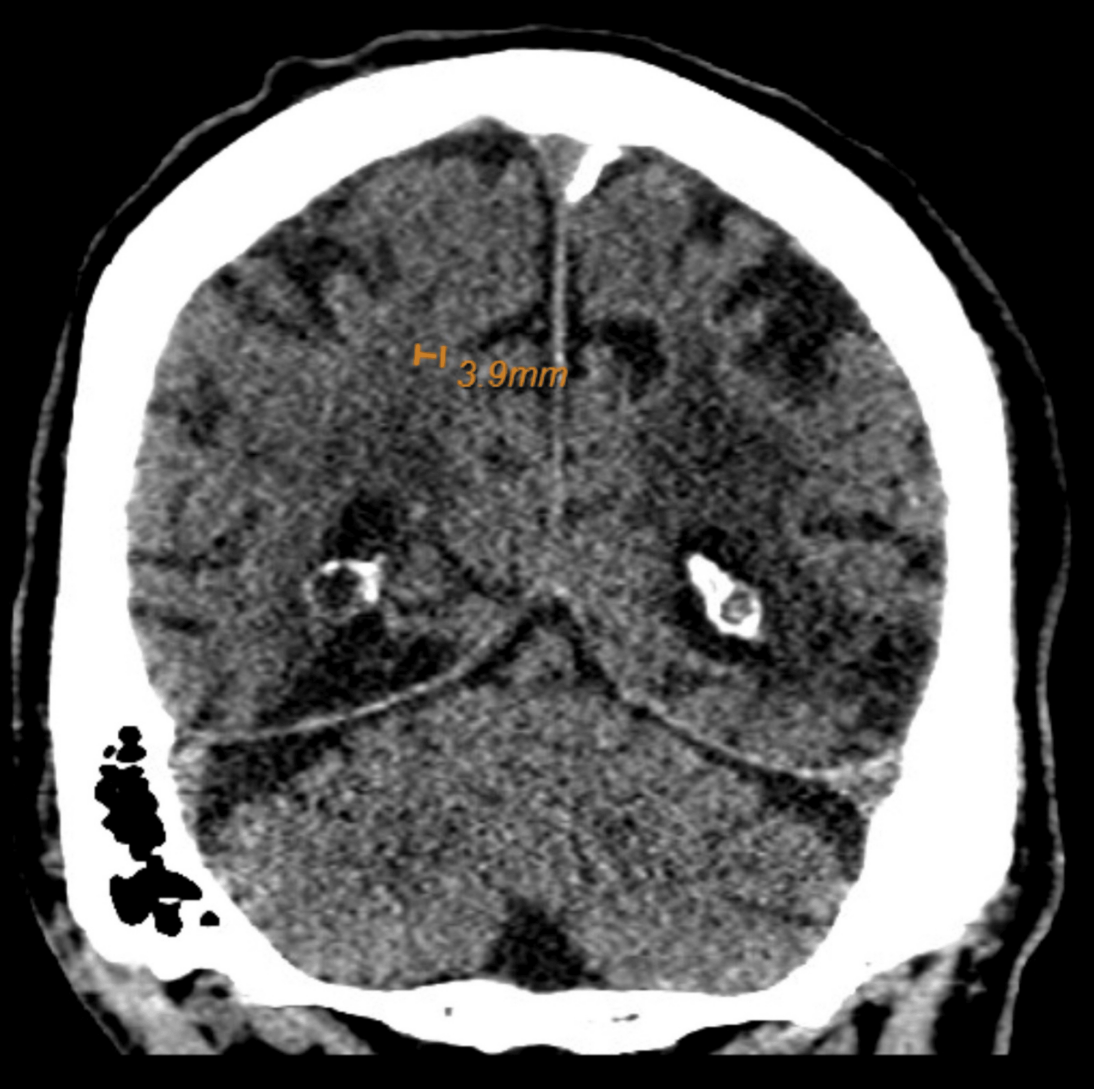
Computed tomography of the brain on day 16 of hospitalization: diminished size of a high right parietal focus of hyperattenuation measuring 4 mm, consistent with the evolution/resolution of parenchymal hemorrhage.

The patient was admitted for the management of extended-spectrum beta-lactamase-producing *Escherichia coli* (ESBL) with a two-week course of 1 g of meropenem every eight hours. Subsequent CT scans of the brain showed a reduction in the size of the hemorrhage, indicative of resolution (Figure 3).

**Figure 4:**
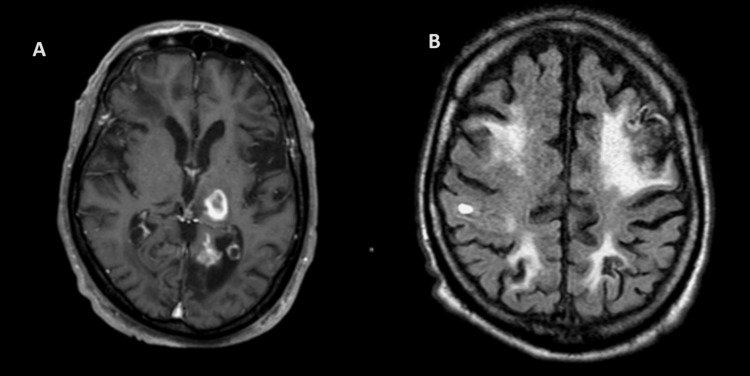
Magnetic resonance imaging of the brain on day 17 of hospitalization. A) T1-weighted post-contrast image in transverse view showing a ring-enhancing lesion in the left thalamus measuring up to 19 mm, with surrounding vasogenic edema; B) FLAIR image in transverse view shows nonspecific, wispy curvilinear enhancement within the bilateral frontal areas and stable enhancement in the left posterior temporal regions of encephalomalacia FLAIR: Fluid-attenuated inversion recovery

Despite the resolution of the abdominal pain, the patient's mental status showed no signs of improvement. A CT scan of the brain was obtained to evaluate the intraparenchymal brain bleed seen on the initial CT scan and the non-improvement of neurologic status. It identified new areas of ring enhancement within the left thalamus, the left thalamic lobe, and the right parietal lobe, with no clarity regarding the inherent nature of the lesions. Additionally, multiple diffusely distributed infarcts were noted. A magnetic resonance imaging (MRI) (Figure 4) obtained to further characterize the findings noted on the CT scan demonstrated multiple ring-enhancing lesions in the left thalamus and non-specific enhancement within the bilateral frontal and left posterior temporal regions of the brain [6,7]. Based on the distribution of the lesions, a cardioembolic source was suspected.

Of note is that the patient did not show any new symptoms, and multiple blood cultures obtained during the hospital course were negative.

**Figure 5:**
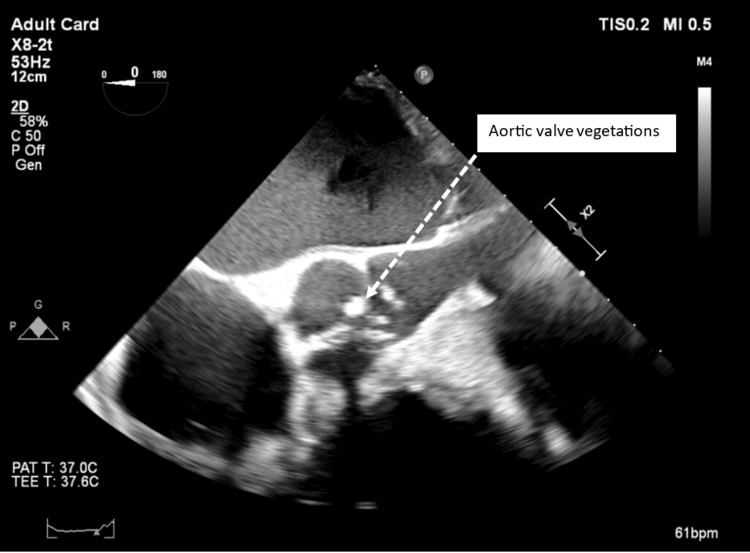
Transesophageal echo obtained on day 21 of hospitalization demonstrating multiple mobile vegetations on the aortic valve.

A transthoracic echocardiogram (TTE) was non-contributory [8]. A transesophageal echocardiogram (TEE) was obtained for further evaluation (Figure 5). It revealed mobile echo densities on the aortic leaflets, likely vegetations, with perforation of the non-coronary cusp causing severe aortic regurgitation, indicative of ongoing infective endocarditis [9]. The primary differential diagnoses at that time included *C. auris* endocarditis versus BCNE. The patient was treated empirically with trough-based dosing of vancomycin and 1 g of meropenem every eight hours for a week while Karius testing, a sophisticated molecular technique used to detect microbial cell-free DNA (cfDNA) in blood samples even when living organisms are no longer identifiable in the blood or when the patient has been on antibiotics, was performed [10]. This test subsequently confirmed the presence of *S. gordonii* in the patient’s blood [11].

The patient was referred for aortic valve replacement but was determined to be a poor surgical candidate. Repeat imaging showed significant improvement in the size of the abscesses, prompting a decision to continue vancomycin with a trough target of 15-20 mg/L and meropenem 2 g every eight hours for a total duration of six weeks. The lesions had decreased significantly by then; however, they remained more than 1 cm in size, due to which antibiotics were continued for two more weeks, resulting in a total duration of 7.5 weeks and a reduction in the size of the lesions to less than 5 mm, with significant improvement in the patient’s neurologic status, at which point the patient was discharged from the hospital.

This case illustrates the complexities associated with the diagnosis and management of BCNE with multiple comorbidities and highlights the importance of advanced imaging and molecular diagnostic techniques in the identification of the culprit pathogen. Through this detailed investigation and tailored treatment approach, the care team aimed to address both the primary infection and its secondary complications, striving to improve the patient's overall health status and cognitive function.

## Discussion

BCNE can arise from various fastidious organisms that are difficult to culture, including the HACEK group, nutritionally variant streptococci, and intracellular pathogens like *Bartonella* species. Fungi such as *C. albicans* and less common ones, such as *Aspergillus* spp., also contribute to CNE and account for approximately 1-2% of the cases. Intracellular pathogens like *Bartonella* species are significant contributors, accounting for 12.4% to 28.4% of CNE cases. *T. whipplei*, although rare, is identified in 0.3% to 3.5% of cases, with higher frequencies (up to 6.3%) when cardiac tissue is available for analysis [12].

*S. gordonii*, a member of the viridans group streptococci, is part of the normal oral flora but can become pathogenic under certain conditions, leading to infective endocarditis, particularly in individuals with predisposing factors such as damaged heart valves, prosthetic devices, or, in this case, a history of ICD placement. The viridans group streptococci, including *S. gordonii*, are known for their ability to adhere to cardiac valves and form biofilms, making infections particularly challenging to treat [13].

The Karius test identifies microbial cfDNA in the bloodstream, providing a comprehensive view of the pathogens present in a patient's system. This allows for rapid and accurate diagnosis of a wide range of infections, even when traditional methods fail. By leveraging the early work from Steven Quake's laboratory at Stanford, the Karius team has overcome the challenge of developing a Clinical Laboratory Improvement Amendments (CLIA)-certified blood test that can detect more than a thousand different pathogens simultaneously within one to two days from a single blood draw. The identification of *S. gordonii* in this patient through Karius testing underscores the value of advanced molecular diagnostic techniques in BCNE [10]. This is particularly crucial in cases where timely identification of the pathogen can significantly influence the treatment strategy and improve patient outcomes [4,14].

The treatment of *S. gordonii* endocarditis typically involves prolonged antibiotic therapy, with penicillin or ceftriaxone being the preferred agents due to their effectiveness against viridans group streptococci. In cases of severe infection, or when the patient has a beta-lactam allergy, vancomycin can be used as an alternative. The combination of antibiotics, such as penicillin and an aminoglycoside like gentamicin, may be employed to enhance bactericidal activity, especially in patients with prosthetic valve infections or in those with more severe presentations. The patient in this case received a combination of vancomycin and meropenem, a broad-spectrum carbapenem, which was empirically started due to the severity of the infection and the initial diagnostic uncertainty [11,15].

Complications of bacterial endocarditis are multiple and can be severe, with septic emboli occurring in 22% to 50% of cases. One particularly serious complication is the development of cerebral abscesses, which occur in approximately 2% to 10% of infective endocarditis cases. These abscesses can lead to significant neurological deficits, seizures, and increased intracranial pressure [16,17].

This case also illustrates the utility of TEE in the diagnosis of endocarditis, particularly in culture-negative scenarios where TTE may be non-diagnostic. Additionally, our case report highlights the growing importance of molecular diagnostic techniques, like Karius testing, in identifying elusive pathogens in complex clinical scenarios. These technologies not only enhance diagnostic accuracy but also allow for more precise and effective treatment strategies [4,10].

## Conclusions

The diagnosis and management of BCNE require a high degree of suspicion and the integration of advanced diagnostic tools. This case demonstrates the critical role of TEE and molecular diagnostics in uncovering and addressing the underlying pathogens in endocarditis, particularly when traditional methods prove unyielding. It also underscores the necessity for a multidisciplinary approach involving cardiologists, cardiothoracic surgeons, neurologists, infectious disease specialists, intensivists, and others, to manage such patients effectively, considering both their cardiac and neurological complications.
